# A novel cluster-tube self-adaptive robot hand

**DOI:** 10.1186/s40638-017-0082-2

**Published:** 2017-12-21

**Authors:** Hong Fu, Haokun Yang, Weishu Song, Wenzeng Zhang

**Affiliations:** Department of Mechanical Engineering, Tsinghua University, Beijing, 100084 China

**Keywords:** Robot hand, Underactuated mechanism, Self-adaption, Cluster-tube grasping

## Abstract

This paper proposes a novel cluster-tube self-adaptive robot hand (CTSA Hand). The CTSA Hand consists of a base, a motor, a transmission mechanism, multiple elastic tendons, and a group of sliding-tube assemblies. Each sliding-tube assembly is composed of a sliding tube, a guide rod, two springs and a hinge. When the hand grasping an object, the object pushes some sliding tubes to different positions according to the surface shape of the object, the motor pulls the tendons tight to cluster tubes. The CTSA Hand can realize self-adaptive grasping of objects of different sizes and shapes. The CTSA Hand can grasp multiple objects simultaneously because the grasping of the hand acts as many grippers in different directions and heights. The grasping forces of the hand are adjusted by a closed-loop control system with potentiometer. Experimental results show that the CTSA Hand has the features of highly self-adaption and large grasping forces when grasping various objects.

## Background

Robot hands have a wide range of uses in the field of robotics. They are used to connect object with robot temporarily and can release the object at the appropriate time. It is one of the important interaction terminals between robots and their outside world.

There are many robot hands mimic structure and configuration of human hands, designed to have multiple fingers and multiple joints in their fingers, for example, the Utah/MIT Dexterous Hand [1], the DLR/HIT Hand II [2], the Gifu Hand II [3] and the Gifu Hand III [4], the Shadow Hand [5], etc. However, these dexterous hands are expensive with complex control and sensing system [6–8].

Since 1970s, a large number of underactuated hands have been developed to highly decrease the difficulty in real-time control of robot hands. These underactuated hands can be easy to control. Some grippers are designed to be self-adaptive, which do not need to know the shapes and sizes of the objects in advance, and do not need sensors to detect positions of objects while grasping. The self-adaptive performance for objects of different shapes and sizes enables the gripper grasp a wide range of objects.

Underactuated hands developed are mainly divided into two kinds: (1) multi-fingered underactuated hands, such as the underactuated prosthetic hand [9], the FRH-4 Hand [10], the tendon-mechanism hand [11], the GR2 Gripper [12], and the SDM Hand [13]. (2) Special underactuated hands without obvious finger, for example, the universal gripper with a spherical structure [14, 15], the adaptive gripper [16] by FESTO company, and the SSA Gripper [17]. These hands are well fitted to the target objects in grasping. However, the two kinds of traditional underactuated hands mentioned above can only grasp one object each time and are sensitive to orientations of objects.

**Fig. 1:**
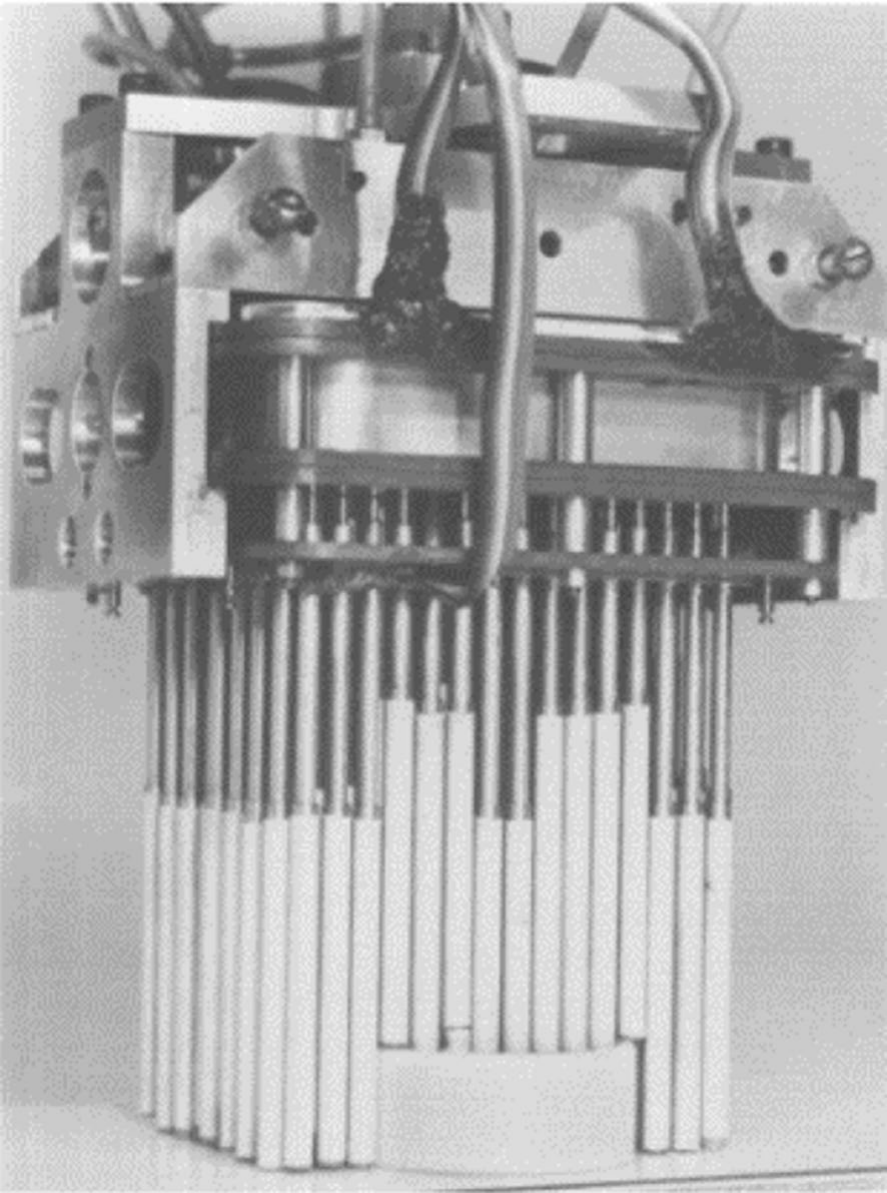
The Omnigripper

**Fig. 2:**
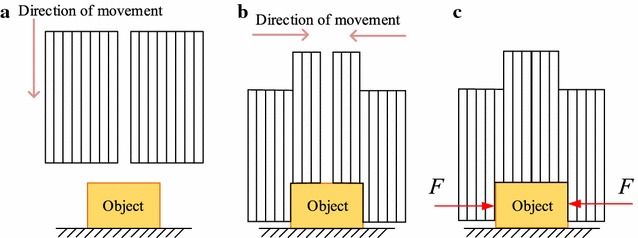
The principle of Omnigripper. **a** Omnigripper approaches object, **b** Omnigripper clamps from two sides, **c** Omnigripper finish grasping

In 1985, Peter B. Scott proposed a general gripper, called Omnigripper [18], as shown in Fig. 1. Many of the independent telescopic rods of the gripper can be freely telescoped when the object is grasped. When grasping, this Omnigripper moving toward an object placed on a support surface like Fig. 2a, then object can squeeze the telescopic rods to slide to the palm. As there are many telescopic rods, different telescopic rods have varying degrees of sliding to the palm, which have relation to the shape of the object, as shown in Fig. 2b. After that, the left and right of the two sets of telescopic rods close and provide clamping force from both sides of the object, as shown in Fig. 2c. This kind of self-adaptation can be well adapted to the size and shape of the object and can provide a large clamping force.

However, there are still shortcomings:

1. *No multi-directional grasping* When the device applies a gripping force to the target object, the gripping force can only be in the moving direction of two sets of rods, resulting in only one-dimensional clamping mode, so the adaptive effect is not ideal. For example, when grasping elongated object whose direction is the same as that of the left and right groups of rods, like the object A shown in Fig. 3, the Omnigripper will fail. What’s more, when grasping object which is completely covered by a set of rods, like the object C shown in Fig. 3, the Omnigripper will fail, too.

**Fig. 3:**
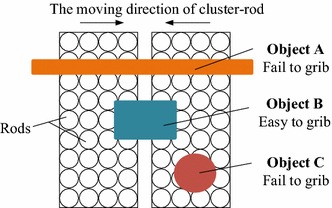
The working principle of omnigripper
2. *High energy consumption* The device has two sets of rods, requiring two mutually moving movable supports (or movement bases), which needs to drive two heavy movement bases to grasp, bringing high energy consumption.

## Design of the CTSA Hand

**Fig. 4:**
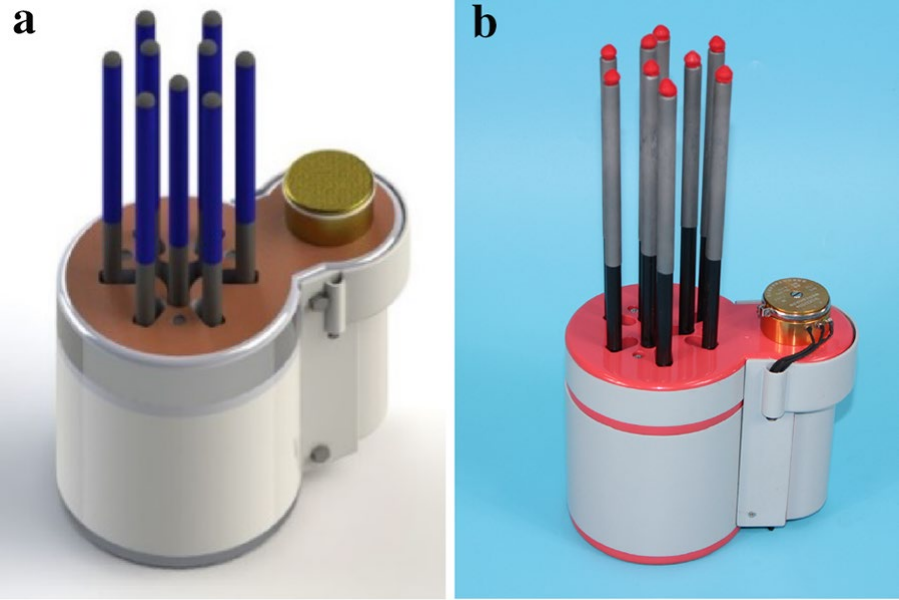
Cluster-tube self-adaptive robot hand. **a** The model of CTSA Hand, **b** the prototype of CTSA Hand

In order to overcome certain deficiency of current ones, this paper proposes a new type of robot hand named cluster-tube self-adaptive robot hand (CTSA Hand), as shown in Fig. 4.

The CTSA Hand is used for grasping objects, capable of self-adapting the sizes and shapes of objects, realizing multi-directional grasping with the following features:

1. Capable of providing grasping forces in multiple directions, making it possible for CTSR Hand to grasp objects with different sizes, shapes and directions.
2. Capable of grasping multiple objects simultaneously because the grasping of the hand acts as many grippers in different directions and heights.
3. Simple structure and low energy consumption.

The following sections describe the composition and working principle of CTSA Hand.

### Composition principle

**Fig. 5:**
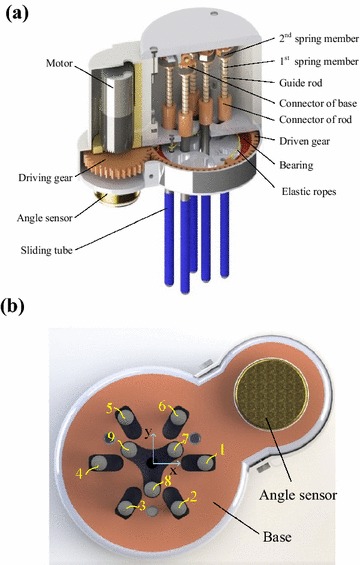
The composition of CTSA Hand. **a** Part section view, **b** bottom view, 1–9: tube1–tube9

The inner structure of CTSA Hand is shown in Fig. 5. It can be divided into three main functional parts—sliding-tube assemblies, transmission system and control system.

#### Sliding-tube assemblies

**Fig. 6:**
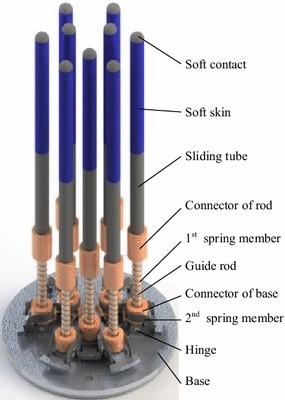
The sliding-tube assemblies of CTSA Hand

**Fig. 7:**
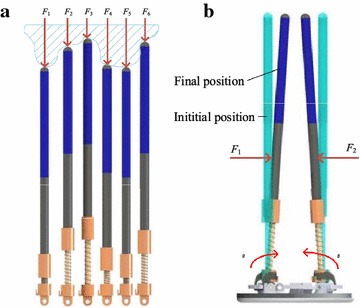
The movement of sliding-tube assemblies. **a** Vertical adaptive ability of sliding-tube assemblies, **b** horizontal adaptive ablility of sliding-tube assemblies

Sliding-tube assemblies are the operating mechanism of CTSA Hand. As shown in Fig. 6, each sliding-tube assembly is composed of a sliding tube, a guide rod, a first spring member, a second spring member and a hinge. Each sliding tube is connected to the guide rod, capable of sliding along the guide rods, as shown in Fig. 7a. Each guide rod is hinged to the base with shaft, capable of swinging in radial direction, as shown in Fig. 7b. Each first spring member is placed between sliding tube and guide rod, and each second spring member is placed on the hinge connected guide rod and base, making sliding tubes have the ability of backing in place.

#### Transmission system

**Fig. 8:**
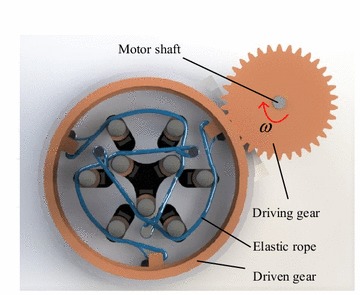
The transmission part of CTSA Hand

The function of the transmission system is to transform the rotation of motor to the gathering of sliding tubes. The transmission part mainly consists of a driving gear, a driven gear and elastic ropes. The driving gear is fixed on the output shaft of motor, and the driven gear is set on the base by bearings. The elastic ropes are fixed between the driven gears and the base: one side of the elastic rope is fixed on the base, while the other side is fixed on the driven gear by passing the sliding tubes. To ensure all sliding tubes are stressed uniformly, three groups of elastic ropes are set in CTSA Hand, the layout of which is shown in Fig. 8.

#### Control system

**Fig. 9:**
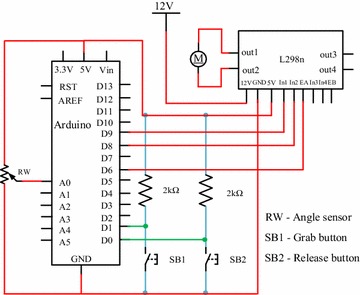
The control circuit of CTSA Hand

The control system adopted Arduino environment, using an angle sensor to monitor driving gear, consequently provide feedback to perform driving or not. The specific principles are as follows: the shaft of the angle sensor is fixed to the driving gear, output real-time angular velocity of the driving gear. When angular velocity deduces to a certain value, which means the CTSA Hand has provided enough grasping force, then the control system terminates the PWM waves input to terminates driving the motor. The control circuit of the Arduino control part is shown in Fig. 9.

### The working principle of CTSA Hand

**Fig. 10:**
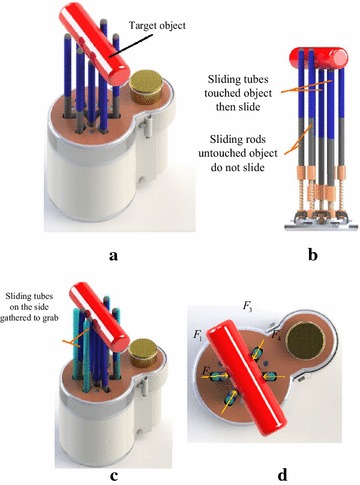
The work process of CTSA Hand. **a** CTSA Hand adapt to the object’s shape vertically, **b** perspective view of a Fig. 10a, **c** CTSA Hand hold the object in all directions from the side, **d** bottom view of Fig. 10c

The working process of the CTSA Hand is shown in Fig. 10. The CTSA Hand approaches target object placed on support surface with the assistance of mechanical arm, and press on the target object, meanwhile the cluster-tube adapt to the object’s shape vertically, as shown in Fig. 10a, b. When the vertical self-adaption is finished, the motor drives the driven gear by the driving gear, causing elastic ropes tense up to gather sliding tubes to central point, as shown in Fig. 10c, d. While the tubes gather, they provide grabbing force in every direction in horizontal plane, resulting in horizontal self-adapting. An angle sensor is adopted to give feedback of the angular velocity of the driving gear, thereby terminates driving the motor at proper time automatically and then realize grabbing. The grasping forces of the hand are adjusted by a closed-loop control system with angle sensor.

## Model analysis

### Analysis and design of gears

For compact structure, we chose the ordinary gear train as the transmission mechanism, and in addition, the ordinary gear train was composed of two gears.

#### Modification of module and teeth number

There is no special requirement for the train wheel drive, so the spur gear is used. Let *z*_1_ and $d_{1}$ be the teeth number and standard pitch circle diameter of driving gear, $z_{2}$ and $d_{2}$ be the teeth number and standard pitch circle diameter of driven gear, $m$ and $a$ be the module and center distance of spur gears. Their relationship is given by the following formula:

1 $$d_{1} = mz_{1} ,$$

2 $$d_{2} = mz_{2} ,$$

3 $$a = (d_{1} + d_{2} )/2.$$

According to the size of the motor and the base, we get the center distance of the gears of the two gears:

4 $$a = 70.5\;{\text{mm}} .$$

In order to compact the structure and the maintenance of the gears, the driven gear needs to be enclosed inside the base, so $d_{2}$ needs to meet the condition:

5 $$d_{2} < D - 2\delta = 94\;{\text{mm}}$$

where *D* is the peripheral diameter of the base and *δ* is the wall thickness of the base.

To meet the strength requirements, we set the module of 1.5, then according to (1)–(5), one can get the following results:

6 $$z_{1} = 34,\;z_{2} = 60,\;d_{1} = 51\;{\text{mm,}}\;d_{2} = 90\;{\text{mm}} .$$

This design result meets the mechanical design requirements.

#### Transmission ratio of the gears

Let $i$ be the transmission ratio of the gears, according

7 $$i = \frac{{z_{2} }}{{z_{1} }},$$

one can get that the gears transmission ratio is 1.77, in line with mechanical design requirements.

### Grasping force analysis

Let $T_{0}$ and $T_{1}$ be the torque of the motor and the output torque of driven gear, respectively. Their relationship is given by

8 $$T_{1} = T_{0} \times i \times \eta$$

where $\eta$ is efficiency of the ordinary gear train. Let $F_{\text{e}}$ be the tensile force of the elastic rope acting on a sliding tube, $N_{\text{tube}}$ be the number of tubes, $N_{\text{tendon}}$ be the number of tendons, then

9 $$T_{1} = \frac{{N_{\text{tube}} }}{{N_{\text{tendon}} }} \times F_{\text{e}} \times \frac{{d_{ 2} }}{2} = 1.5F_{\text{e}} \times d_{2} .$$

**Fig. 11:**
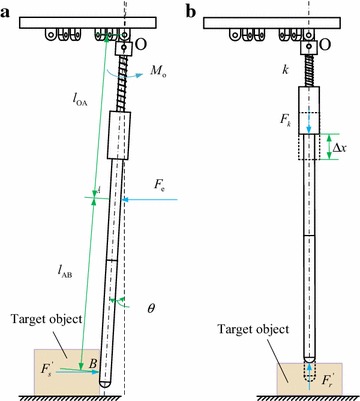
Analysis of the force acting on the sliding tube. **a** Sliding tube touches the object from the side, **b** sliding tube touches the object from the top

We simplify the force of one sliding tube as shown in Fig. 11. Let $\theta$ be the swing angle of a siding rod, $F_{\text{s}}^{\prime }$ be the squeeze force of the target object acting on a sliding tube from the side, and $M_{\text{o}}$ be the torque provided by a second spring member, then

10 $$F_{\text{s}}^{\prime } \times l_{\text{OB}} \times \cos \theta + M_{\text{o}} = F_{\text{e}} \times l_{\text{OA}} \times \cos \theta$$

where $l_{\text{OA}}$ and $l_{\text{OB}}$ are the distance from acting point of elastic rope and acting point of the target object to the hinge point *O*, respectively. Let $F_{\text{r}}^{\prime }$ be the resistance of the target object acting on a sliding tube in the vertical direction, *k* be the elastic coefficient of the first spring member, and $\Delta x$ be the distance of a sliding tube slides, then

11 $$F_{\text{r}}^{\prime } = k \times \Delta x.$$

From (7) to (10), one can get the following conclusion: The squeeze force of a sliding tube acting on the target object is

12 $$F_{\text{s}} = \frac{{2i\eta l_{\text{OA}} T_{ 0} }}{{3l_{\text{OB}} d_{ 2} }} - \frac{{M_{\text{o}} }}{\cos \theta }.$$

Then, let m be the number of tubes squeezing object, *n* be the number of tubes touching the object from above, $\mu$ is the coefficient of friction between the target object and the surface material of sliding tubes, and *F* be the grasping force the CTSA Hand provided, then

13 $$F = \sum\limits_{i = 1}^{m} {\mu F_{{{\text{s}}i}} } - \sum\limits_{j = 1}^{n} {F_{{{\text{s}}j}} } ,$$

From (11) to (13), one can get

14 $$F = \mu \sum\limits_{i = 1}^{m} {\left( {\frac{{2i\eta l_{\text{OA}} T_{ 0} }}{{3l_{\text{OB}} id_{ 2} }} - \frac{{M_{{{\text{O}}i}} }}{{\cos \theta_{i} }}} \right)} - \sum\limits_{j = 1}^{n} {k\Delta xj} .$$

In order to achieve a large grasping force, one can choose a motor which can provide large torque, reduce the driven gear diameter, increase the transmission ratio of the gear train, increase $l_{\text{OA}}$ and reduce $l_{\text{OB}}$, choose a surface material to increase the coefficient of friction between the sliding tube and the target object, reduce the elastic coefficient of the first spring member and the second spring member.

## Experiments

### Experiments of verifying adaptation

**Fig. 12:**
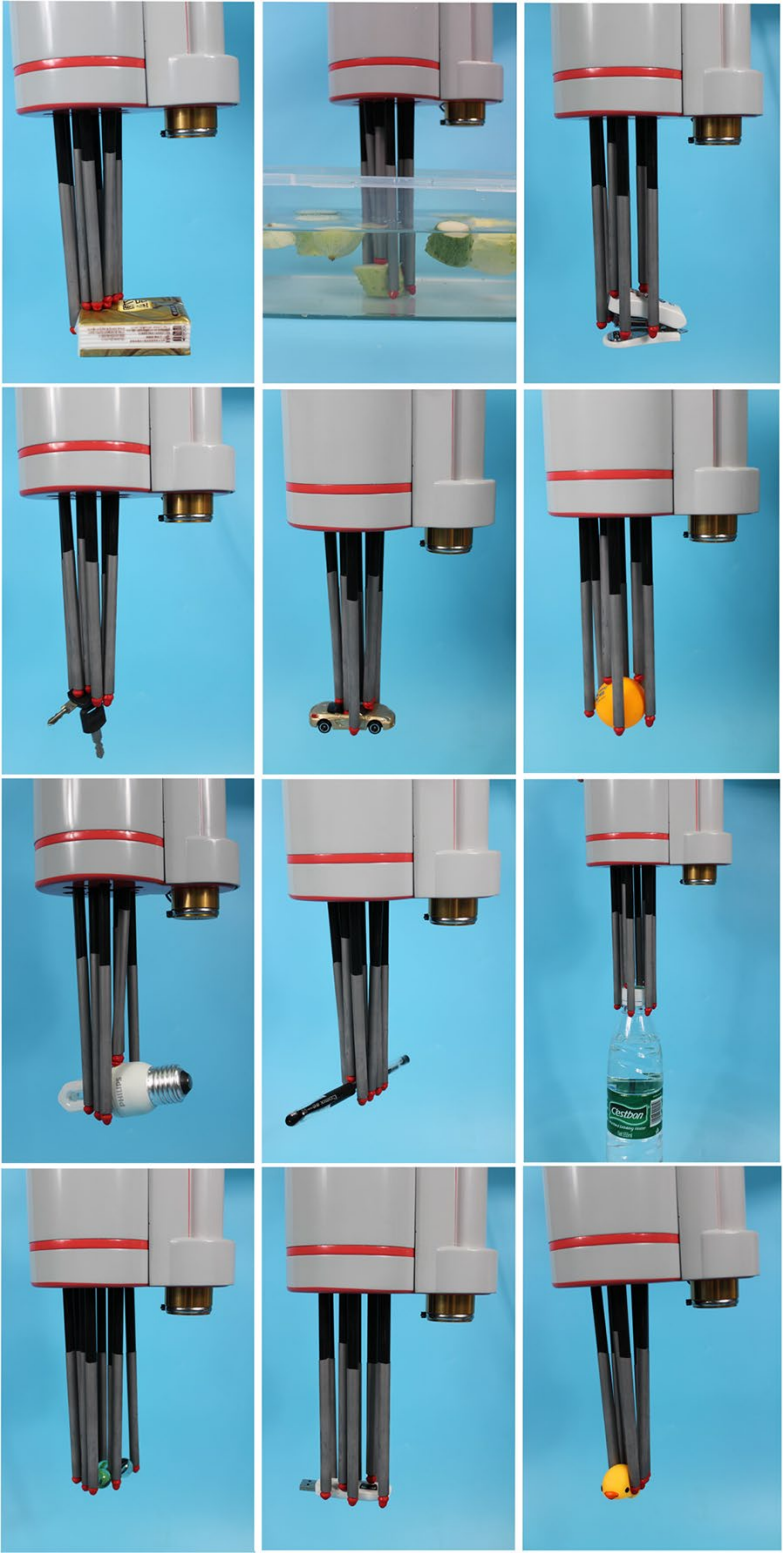
The CTSA Hand grasping different objects

The grasping experiment is conducted to verify the adaptation and stability of CTSA Hand. Figure 12 shows CTSA Hand grab a variety of objects with different shapes and sizes well. The experiment shows:

1. The CTSA Hand can adapt to the shape and size of different objects well.
2. The CTSA Hand has the features of highly self-adaption through the vertical and horizontal adaption.
3. The grasping reliability of the CTSA Hand is high.

### Experiments of gripping performance

#### Design experiment

This experiment is conducted to explore how the size of the object affects the grasping force of CTSA Hand. We used 3D printer to produce cuboids with different sizes. The experimental idea is to measure the maximum grasping force that CTSA Hand can provide for different cuboids and then explore how the size of cuboids affects the grasping force. So we designed the experimental steps as follows:

1. Tie the rope to a cuboid of a particular size.
2. Place the cuboid with the rope on the table and drive CTSA Hand to grab it.
3. Use a dynamometer to pull the rope tied to the cuboid slowly until the cube leaves CTSA Hand.
4. Record the peak of the dynamometer in the process.

In this experiment, the peak of the dynamometer is the maximum grasping force that CTSA Hand can provide for the certain cube.

In addition, in order to make the collected data more reasonable, we collect the data as follows: For a cube with a specific size, we repeat the above experiment steps three times and then get three data points; the average of these three data points is the maximum grasping force that CTSA Hand can provide.

#### Experimental data processing and analysis

**Fig. 13:**
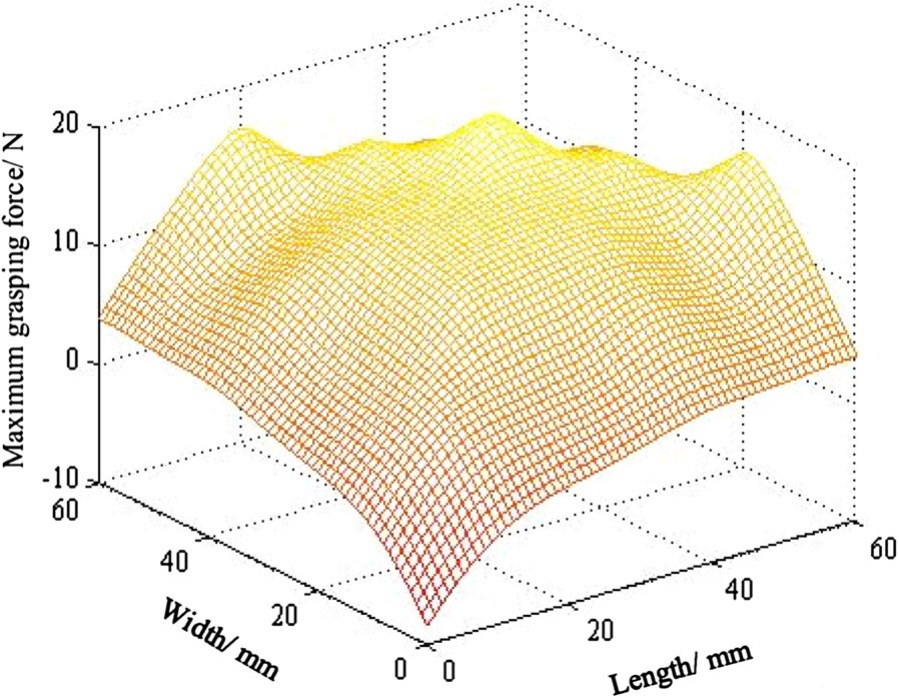
The relationship between the maximum grasping force and the cross-sectional size of cubes in the same height

To explore the effect of cross-sectional dimensions of the cube on grab performance, we make 25 cubes that have the same height, different cross-sectional dimensions using 3D printing. Then, we get the different crawl data of 25 different sizes of cubes through the above method, and then use the mathematical analysis tool to fit the experimental data, and finally get the result shown in Fig. 13. In Fig. 13, the maximum grasping force is negative when the size of cuboid is too small, which is caused by mathematical tools. Here, a negative value indicates that the CTSA Hand cannot succeed in grabbing object.

**Fig. 14:**
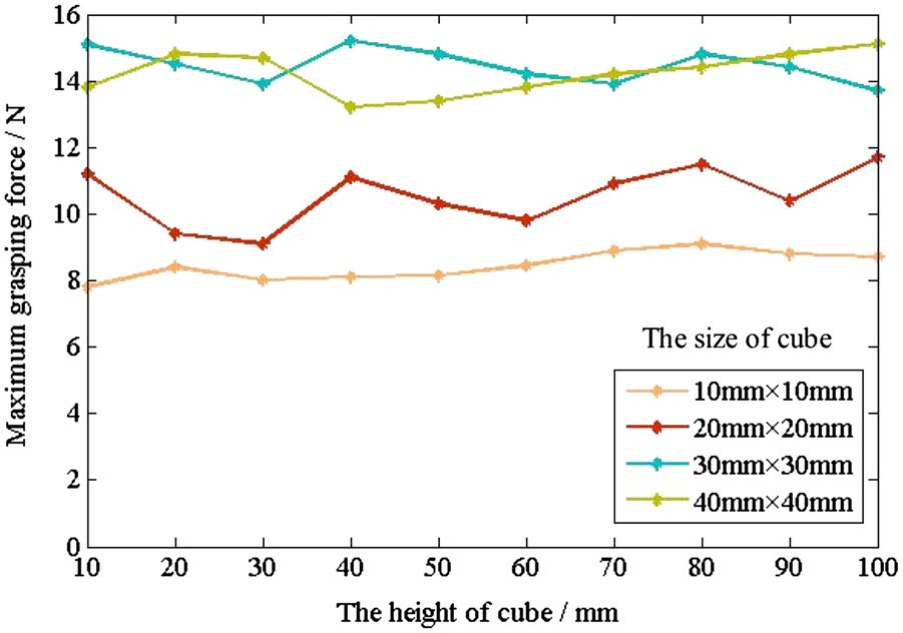
The relationship between the maximum grasping force and the height

In addition, to explore the effect of cube height on grip performance, we created four sets of cubes each containing 10 cubes with the same cross-sectional dimensions and different heights. Then, we get the different crawl data of the four sets of cubes through the above method and then use the mathematical analysis tool to fit the experimental data and finally get the result shown in Fig. 14.

In Figs. 13 and 14, the unit of maximum gripping force is Newton, and the unit of height, width and length is millimeter.

From the results shown in Figs. 13 and 14, we can get the following conclusions:

1. The maximum grasping force of CTSA Hand is changing with the cross-sectional size of the target object and almost no changing with the height of the target object.
2. When the sizes of cuboids are moderate, the gasping force of the hand is large, and the degree of influence by the cross-sectional size is small.
3. When the sizes of the cuboids are too small or too large, the grasping force of the hand is small, even fail to grab.

**Fig. 15:**
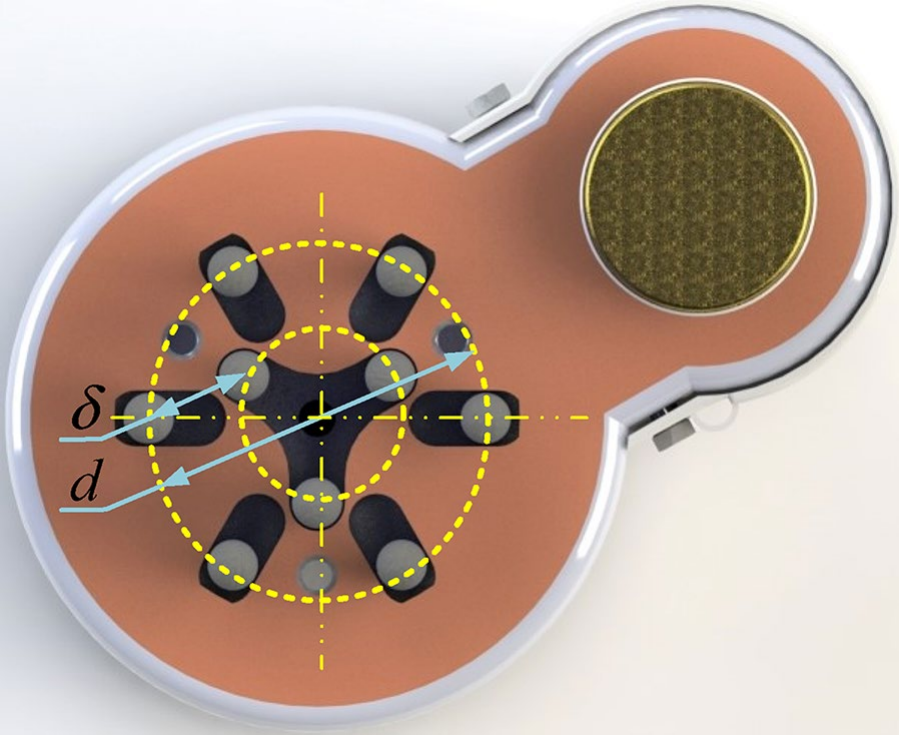
Size *d* and size *δ* in CTSA Hand

After analysis, the phenomenon that the maximum grasping force suddenly become small when the cross-sectional sizes of cubes become too small or too large, is caused by restrictions of *d* and *δ*, where *d* is the maximum internal radius of cluster-tube and *δ* is the minimum distance between tubes, as shown in Fig. 15. The size *d* affects CTSA Hand’s grasping force: The larger the size *d*, the greater the size of the object that CTSA Hand can crawl, and the greater the grasping force for large objects. The size *δ* affects the robot’s grasping force: The smaller the size *δ*, the smaller size of the object that CTSA Hand can crawl, and the greater the grasping force for small objects.

#### The results of experiments

Through the analysis of experimental data, we can get that CTSA Hand has the advantages of large grasping force and high stability when grasping a variety of medium-sized objects. However, there are still some shortcomings such as small grasping force when grasping too small or too large objects. To solve the current problems, we will reduce the size *δ* and add more sliding-tube assemblies to increase the size d in our future design.

## Conclusions

In order to overcome some shortcomings of the existing robot hands, this paper proposes a novel cluster-tube self-adaptive robot hand (CTSA Hand).

The CTSA Hand consists of a base, a motor, a transmission mechanism, multiple elastic tendons and a group of sliding-tube assemblies. Each sliding-tube assembly is composed of a sliding tube, a guide rod, two springs and a hinge. When the hand grasping an object, the object pushes some sliding tubes to different positions according to the surface shape of the object, the motor pulls the tendons tight to cluster tubes.

The CTSA Hand has the features of highly self-adaption and large grasping force when grasping various objects. The CTSA Hand has the advantages of easy control, simple structure, low cost and high reliability of grasping. Therefore, it has the value and potential of mass production and large-scale applications and can be widely used in the fields of service robotics, industrial production and scientific research.
